# An Act to Provide for the More Efficient Granting of Diplomas by Medical Colleges

**Published:** 1881-11

**Authors:** 


### AN ACT

To provide for the more efficient granting of diplomas by medical colleges.

SECTION 1. Be it enacted by the General Assembly of the State of Georgia, and it is hereby enacted by the authority of the same, That, from and after the passage of this Act, it shall be unlawful for the faculty or officers of any medical college in the State of Georgia to grant or issue a diploma to any student of medicine, or other person, unless said student or other person shall have attended two or more full courses of study in some regularly chartered medical college in good standing, and shall have submitted to and passed a creditable examination by the faculty or professors of said college, upon all the branches usually taught in medical colleges

SEC. 2. Be it further enacted, That if the faculty or officers of any medical college in this State shall violate any of the provisions of the preceding section of this Act, he or they shall be subject to a fine of five thousand dollars, said fine to be collected out of the property of any or all of said faculty or officers of said college. The fine, when collected, shall be paid the one-half to the person, persons or corporation giving the information, the other half into the county treasury, to be used for educational purposes only.

SEC. 3. Repeals conflicting laws.

Approved, September 27th, 1881.

---

